# A concentric circles view of health data relations facilitates understanding of sociotechnical challenges for learning health systems and the role of federated data networks

**DOI:** 10.3389/fdata.2022.945739

**Published:** 2022-09-16

**Authors:** Richard Milne, Mark Sheehan, Brendan Barnes, Janek Kapper, Nathan Lea, James N'Dow, Gurparkash Singh, Amelia Martín-Uranga, Nigel Hughes

**Affiliations:** ^1^Wellcome Connecting Science, Cambridge, United Kingdom; ^2^Kavli Centre for Ethics, Science and the Public, Faculty of Education, University of Cambridge, Cambridge, United Kingdom; ^3^Ethox Centre, Nuffield Department of Population Health, University of Oxford, Oxford, United Kingdom; ^4^Oxford National Institute for Health and Care Research (NIHR) Biomedical Research Centre, Oxford University Hospitals Trust, Oxford, United Kingdom; ^5^European Federation of Pharmaceutical Industries and Associations, Brussels, Belgium; ^6^Estonian Chamber of Disabled People/European Patients Forum, The Estonian Inflammatory Bowel Disease Society, Tallinn, Estonia; ^7^Institute for Innovation Through Health Data (i-HD), Gent, Belgium; ^8^Academic Urology Unit, University of Aberdeen, Aberdeen, United Kingdom; ^9^Janssen Research and Development, Titusville, NJ, United States; ^10^Farmaindustria, Madrid, Spain; ^11^Janssen Research and Development, Beerse, Belgium

**Keywords:** consent, data, trust, federated data access, distributed data access, ethics

## Abstract

The ability to use clinical and research data at scale is central to hopes for data-driven medicine. However, in using such data researchers often encounter hurdles–both technical, such as differing data security requirements, and social, such as the terms of informed consent, legal requirements and patient and public trust. Federated or distributed data networks have been proposed and adopted in response to these hurdles. However, to date there has been little consideration of how FDNs respond to both technical and social constraints on data use. In this Perspective we propose an approach to thinking about data in terms that make it easier to navigate the health data space and understand the value of differing approaches to data collection, storage and sharing. We set out a *socio-technical* model of data systems that we call the “Concentric Circles View” (CCV) of data-relationships. The aim is to enable a consistent understanding of the fit between the local relationships within which data are produced and the extended socio-technical systems that enable their use. The paper suggests this model can help understand and tackle challenges associated with the use of real-world data in the health setting. We use the model to understand not only how but *why* federated networks may be well placed to address emerging issues and adapt to the evolving needs of health research for patient benefit. We conclude that the CCV provides a useful model with broader application in mapping, understanding, and tackling the major challenges associated with using real world data in the health setting.

## Background

The large-scale use of real world data (RWD) is central to hopes for learning health systems (Krumholz, 2014). However, efforts to realize these hopes face challenges associated with the complex systems that support health data collection, sharing and use. Some of these challenges can be considered primarily “technical”–for example related to the ability to manage the security of health data, or deal with multiple and potentially incompatible data formats or models (Curtis et al., 2012; Hripcsak et al., 2015). Others are often considered “social,” “ethical” or “legal,” notably how one ensures that adequate informed consent to data use, maintains public trust in data systems or meets legal and regulatory requirements (Corrigan, 2003; Carter et al., 2015; Aitken et al., 2016).

Resolving either technical or social challenges is a complex endeavor. This is compounded by the fact that on closer inspection, distinctions between these types of challenge can often be difficult to tease apart (Wan et al., 2022). For example, public trust is affected by the success (or particularly the failure) of data security architectures, while the co-existence of multiple data formats, and decisions about which data are relevant to collect, reflect the social, political and economic history that has shaped the development of health data systems and their technical standards (Leonelli, 2019).

In this paper, we argue that this socio-technical intricacy presents a significant problem for the future of learning health systems. Specifically, the tangle of technical and legal standards, ethical rules–including informed consent mechanisms - and social norms can be overwhelming for individuals and organizations attempting to navigate the health data space and paralyzing for health data initiatives. Instead, we suggest a need for tools for thinking and understanding data and its research use in simpler terms. To that end, we set out a socio-technical model of data systems that we call the “Concentric Circles View of data-relationships” (CCV) and describe how it can be used to conceptualize some key challenges associated with health data.

We illustrate how the CCV can be used to examine the potential of one proposed socio-technical solution to these challenges, that of Federated Data Networks (FDNs). FDNs are increasingly recognized as a means of meeting the challenge of bringing together differently located, diverse data sets to allow research without violating local norms, values, and governance arrangements. We suggest that the CCV allows us to understand not only how but why FDNs are well placed to address both “technical” and “social” issues associated with health data. The further elaboration of the CCV may have broader application in mapping, understanding, and tackling the major challenges associated with using RWD in the health setting.

## The concentric circles view: Local, people-centered data relationships

The approach to data relationships we propose starts from two premises. The first is that regardless of its form, content or purpose, all data are local; they have a context, and understanding this context is crucial to practicing responsible big data research (Zook et al., 2017). They have a provenance–they are produced in a specific context, embedded within a particular scientific, ethical political and social milieu (Parry and Greenhough, 2018). They also have contexts of sharing, use and dissemination, which may be the same or different. Contexts of both production and use may thus differ in their material and technical composition and in their social organization and meaning. For example, a local primary care practitioner can send a summary and referral to a hospital specialist as a part of providing care for the patient without specific consent. Alternatively, patients attending the local hospital might agree to allow their data to be used for research. This arrangement might preclude access by commercial institutions to treatment level data but allow access to anonymised data. In a final example, patients admitted to a large university hospital might agree to access of treatment level data by commercial institutions, for instance with certain restrictions, but these institutions must be based in the same country.

The second is that health data are also about particular people–they ultimately relate to a quality of an individual or their interaction with health and research systems. This personal relationship is fundamental to value of data and the social and technical architecture which does the work of connecting or disconnecting data from people–for example by protecting general practice datasets *in situ* or by aggregating, de-identifying or anonymising to allow wider use.

One instructive–if simplified - way of representing and thinking through these relationships can be in terms of a series of concentric circles, the **CCV**. Each circle in this model represents an idealized representation of the socio-technical arrangement that frames the relationship between the data subject and the people and institutions, tools, laws, and ethical frameworks involved in data production and use. Reflecting our second premise, the CCV is centered on the data subject–the person, patient, or research participant to whom data applies and who occupies the center of the circles. At this central point, the social and technical arrangements that are in place aim to ensuring that data retain a direct relationship with the individual.

Outer circles in the model reflect different arrangements of the data relationship. In each circle, the context of data use (and the production of new forms of data) involves putting in place a different set of tools, regulations and processes that treat data in different ways. These circles may not present in this way for any specific individual, and individuals will differ in terms of who 'stands' in which circle for them. Data users may also sit in different circles at different times for different individuals – a pharmaceutical company for example, may fulfill the requirements of an inner circle in conducting a clinical trial, while sit in an outer circle when drawing on aggregated genomic data in the process of drug discovery.

**Figure 1:**
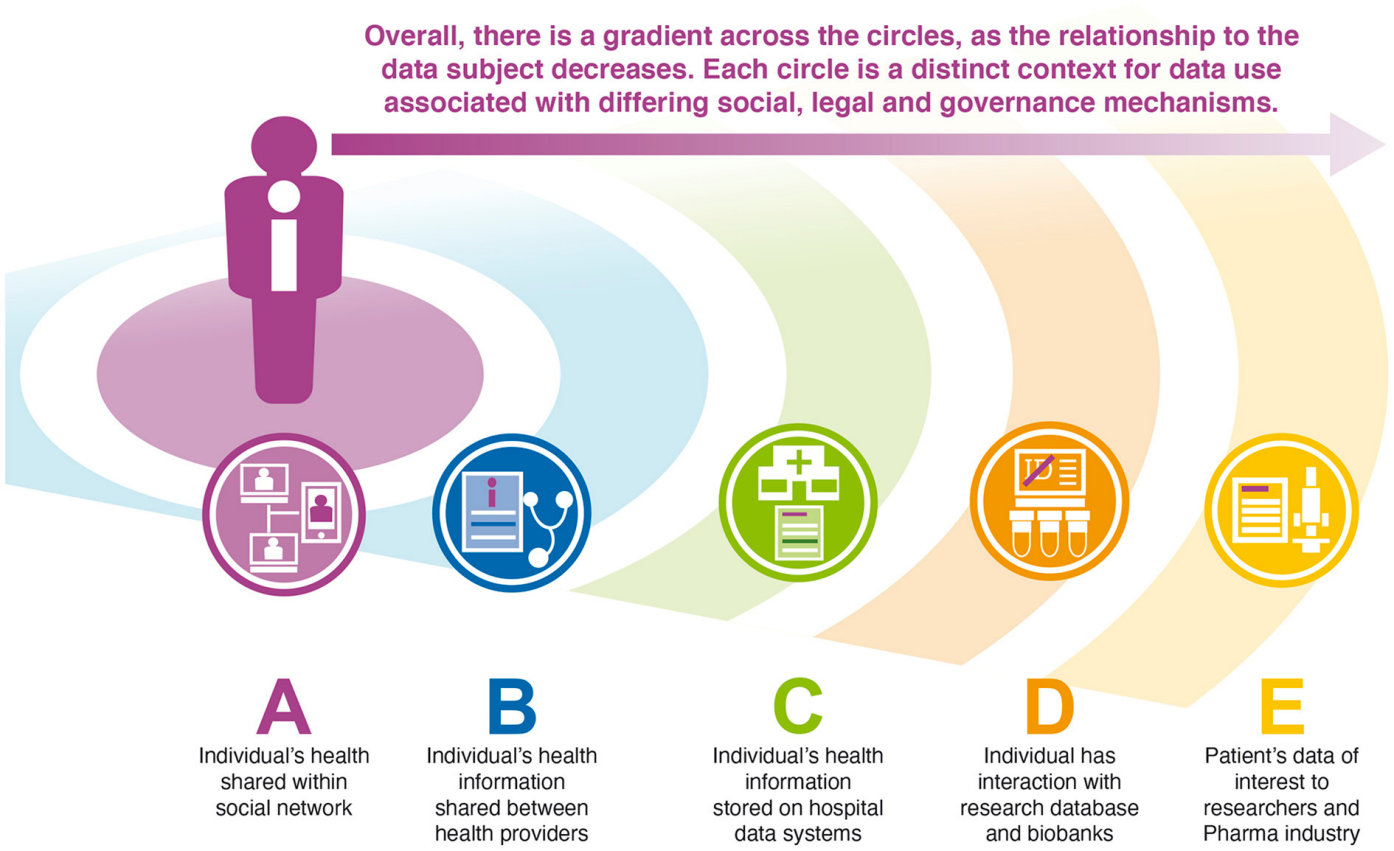
The Concentric Circles View of a possible arrangement of data relationships for a single individual. In the proposed model, the initial circle **(A)** is the most intimate to the individual, and here involves the direct sharing of information within an individual's social network. Data related to this individual are also shared between health providers **(B)**, stored on hospital data systems **(C)** and in research studies in which they participate **(D)** and used, in anonymised form by other researchers and the pharmaceutical industry, for example in drug discovery research **(E)**. Each of these contexts involves a distinct social, legal, ethical and technical configuration.

One possible configuration of the circles in the CCV is shown in Figure 1, and represents one possible arrangement of data users in relation to a single individual. While the content and configuration of the CCV will be individual, representing the relationships in this way can be understood as a tool for thinking with, a heuristic that offers a way of simplifying and depicting the complexity of the health data space. The use of the CCV as a thinking tool may enable a consistent conceptualization of the socio-technical system that enables data use, and as such allows a clearer understanding of the strengths and weaknesses of different approaches to health data sharing.

## Using the CCV to conceptualize data relations

How we think about the ethical, legal, and technical issues associated with data use flows from the relationships within each circle and changes as one moves through the circles. Relationships closer to the data subject tend to prioritize security over sharing, driven primarily by duties of confidentiality and relations of trust. These duties and relations, and the associated access controls change as one moves out through the circles. This change is both quantitative, in terms of intensity or scope, and qualitative, in the nature of the data and controls, and differences in duty and trust.

Overall, as one moves “outwards” through the model and becomes further “removed” from the data subject, data become less granular and less easily identifiable. The model helps to show, however, how and why making data less granular requires work. This work is structured by different technical systems for data sharing (e.g., sharing of anonymised or aggregated data or the construction of trusted research environments), legal and ethical frameworks (e.g., associated with large scale public health research and policy), and social relationships [e.g., where trust is placed and the balance between relations of trust and reliance (Sheehan et al., 2020)]. Moving “inwards” from an outer circle to a position closer to the data subject again requires work, for example building systems for privacy protection associated with more identifiable data, obtaining direct consent from the data subject and establishing closer relationships of trust. The changes associated with moving between circles can be illustrated through specific examples associated with the ethical, legal and social contexts of data collection and use including trust, informed consent, and public and patient involvement.

Taking trust first, the inner circles are characterized by direct interactions between individuals and those who are using “data,” including the usual sharing of information within families, or between doctors and patients. “Data collection” here may not necessarily be considered as such, even when it results in entries in a general practice data system, and is primarily interpersonal, grounded in the relationship between an individual data subject and other known individuals (Sheehan et al., 2020). In contrast, relationships with a biobank or a hospital may involve a more generalized type of trust between an individual and the institution or system or a set of governance arrangements – for example the NHS – and/or a reliance on technical or legal systems that protect health data (Lipworth et al., 2009; Gilbar, 2012; Steedman et al., 2020). Importantly, the trust built on these relations is not fixed and immutable but complex and changeable: the amount of information a person is prepared to share with others will vary, as will their comfort in sharing personal information with healthcare professionals. This may be affected by an awareness of how individuals or organizations interact with those in other circles (as illustrated by the impact of perceived commercial motivations or involvement on trust in public sector data collection), and the systems involved in regulating or governing these interactions (such as the strength of sanctions associated with breaches of trust) (Milne et al., 2021).

The question of consent is a particularly useful example and illustrates that not only are the changing relationships between the data subject and each circle important in understanding the conditions that create a specific context for health data, but that the relationship *between* these contexts provides a means of understanding many of the ethical, social, and technical challenges associated with using health data. Overarching legal and policy requirements within geographical jurisdictions, such as General Data Protection Regulation (GDPR), implemented in 2018 in Europe, the Consumer Privacy Act in California, 2018, or the draft PRC Personal Information Protection Law (PIPL) in China, all rely on concepts of consent. The form and content of consent for data collection though differs across the health data ecosystem represented by the CCV. Reflecting our premise related to the local provenance of data, consent in one circle does not necessarily allow for data to move, and may not allow it to move *between*, rather than *within* circles.

This points to the specific work done by those forms of consent that *do* allow for data to move between circles, and how they draw attention to the additional governance and/or regulatory caveats associated with crossing socio-technical contexts. For example, sharing of data generated in clinical interactions (B) to research organizations situated in an outer circle may require specific legal provisions, such as de-identifying the data by removing the connections that maintain its relationship with the individual (Gilbar, 2012). In the case of biobanks (D), the initial and often broad consent process may facilitate the collection of data or samples, but further sharing of or access to these data may involve governance mechanisms, such as data access committee approvals, acting on behalf of the institute and data of which they are a custodian (O'Doherty et al., 2021). In contrast, the sharing of anonymised summary level data for genome-phenome analyses Genome Wide Association Studies (GWAS) may occur through derestricted databases (Wan et al., 2022).

A final example of the changes associated with the move between circles is the appropriate form of inclusion and representation of public, patient or participant perspectives in decisions about data collection and use. The CCV allows a conceptualization of the nature of public involvement and its ability to legitimately represent the interests of patients, the concerns of publics and potential tensions between them. Such representation is increasingly common, but there is a lack of clarity about its appropriate form and scope across complex health data systems (Erikainen et al., 2020). In inner circle data relationships, in which direct connections exist between data and the patient, involvement ordinarily means the patient themself being involved in the decision about how data are produced, used and accessed (Samuel and Farsides, 2018). However, this direct involvement is neither practical nor necessarily appropriate in circles further from the core. Thus, for a biobank or research database (D) representation may focus on the population or community represented in the dataset, in the form of a community advisory board, or the involvement of a patients' organization – and ensuring that such boards are legitimately able to represent broader community perspectives (Strauss et al., 2001). At the extreme, where data may be anonymised or aggregated and have little or no remaining connection to either identifiable individuals or groups, the appropriate form of representation might be that of a wider public consultation to enable the alignment of data access and use within relevant societal values (UK Biobank Ethics and Governance Council, 2009), or simply a reliance on the democratic legitimacy of decisions about data sharing.

In summary, the CCV approach aims to show that the contexts in which data are collected and used, and the relationship between these contexts and the data subject, can be delineated by their social, ethical, legal, and technical qualities. An awareness of these contexts, we suggest, can help to understand the work involved in moving or sharing data, and capture the value of frameworks that maintain the socio-technical integrity of these contexts, while allowing these data to be accessed to achieve the maximal clinical and societal value. As we discuss in the following section, this awareness helps us to understand why FDNs are a promising approach to constructing data architectures for learning health systems.

## Maintaining context integrity

The challenge for data-intensive health systems is to use large volumes of relevant specified data without violating the rules and norms associated with the context in which data are generated and stored. When research requires working outside a particular “circle” and the associated technical, ethical, legal, or social arrangement, this challenge can be daunting, and in some situations, for example in international data sharing where there is a lack of harmonization, overwhelming (World Economic Forum, 2020).

Maintaining the integrity of a circle is thus a crucial challenge for health data initiatives. Two broad approaches to this can be delineated. The first involves attempting to bring all data users within one circle, through establishing a shared socio-technical system. For example, this might be achieved by bringing data users closer to the data subject, into an inner circle and relations of trust in individuals or institutions, specific consent and direct individual, public or patient involvement, and technical systems that emphasize privacy and enabling consent. There are, indeed technological strategies which endeavor to achieve this approach, notably in the form of dynamic consent (Kaye et al., 2015; Ploug and Holm, 2016), but the scale of the work involved for both data users and data subjects makes it is unclear whether these are workable in practice and indeed, given the available alternatives, whether it is required from an ethical standpoint (Sheehan, 2011; Manson, 2019; Sheehan et al., 2019). An alternative approach is to bring all data use in a more distant, but still shared position in relation to the data subject through the construction of a large database (or data lake). Here, data are held in one large repository and shared with researchers according to pre-specified rules. While sharing the goal of consolidating the data context, the nature of consent, trust, and involvement differs from the first case – in this scenario an initial interaction with the data subject might establish broad consent, in part on the basis of an individual's trust in the institution and system (Hansson, 2005), and supported by the processes of governance that determine who has access to data and to what extent, potentially informed by participant or community involvement (Erikainen et al., 2020).

The drawback of this kind of centralized arrangement comes from the diverse existing approaches that relate to the different prior positions in the CCV. Different data contexts have often divergent histories and traditions of governance and regulation, different relationships to medical research and medical research institutions and make different judgements about trade-offs between privacy, confidentiality, and the benefits of large-scale data-based research. These differences could mean that bringing data users into a common position in relation to the data subject may effectively mean starting again with consent and data collection (Rieke et al., 2020).

As a result, large centrally held databases can struggle to address this diversity, requiring considerable work to coalesce governance and to come to act as a custodian of the data. In contrast, the appeal and the opportunity associated with FDNs can be understood in terms of their ability *to take variance into account* and to harmonize rather than consolidate. FDNs are characterized by a socio-technical framework for the sharing of resources and the ability to query data remotely by way of an interface, with data remaining local. FDNs can be quite specific in their intent, such as the FDA's Sentinel initiative or the proposed DARWIN EU network of the European Medicines Agency for regulatory scientific purposes. Conversely, generic FDNs, often disease and therapeutic area agnostic, can meet wider scientific requirements for academic or commercial use, for example in the EU's Beyond 1 Million Genomes Initiative (Saunders et al., 2019). Table 1 outlines large-scale international FDNs.

**Table 1:** Examples of international federated data networks for health research.

**FDN**	**Status**	**Location**	**Data harmonization**	**Purpose**	**More information**
Sentinel	Regulatory authority	United States	Common data model	Disease agnostic. In-market safety and efficacy analysis	https://www.fda.gov/safety/fdas-sentinel-initiative
DARWIN EU	Regulatory authority (planned)	European Union	Common data model	Disease agnostic. In-market safety and efficacy analysis	https://www.ema.europa.eu/en/about-us/how-we-work/big-data/data-analysis-real-world-interrogation-network-darwin-eu
European Health Data & Evidence Network (EHDEN)	Innovative Medicines Initiative 2 project	European region	Common data model (OMOP)	Disease agnostic. Large-scale real world research, R&D and education	https://ehden.eu
PIONEER	Innovative Medicines Initiative 2 project	European region	Common data model (OMOP)	Prostate cancer. Large-scale real world research, R&D and education	https://prostate-pioneer.eu/
TriNetX	Commercial	Global	Common data model	Disease agnostic. Study design, trial operations, and post-approval research	https://trinetx.com/
Observational Health Data Sciences & Informatics (OHDSI)	Research	Global	Common data model (OMOP)	Disease agnostic. Large-scale real world research, R&D and education	https://ohdsi.org

Within an FDN, the contexts in which data are held – the Data Partners – can be diverse and situated across the circles of the CCV, from hospitals and hospital networks to claims databases, national datasets, and regional registries. A process of data harmonization using, for instance, a common data model, allows for a distributed model of querying *via* standardized analytical tools. This reduces the need for ongoing data curation on a per study basis. The use of catalogs describing diverse data sources, alongside the adoption of FAIR data principles (Findable, Accessible, Interoperable, and Reusable) enhance interoperability and reusability of source RWD (Weeks and Pardee, 2019). Results are aggregated, while ensuring local technical and governance requirements remain of primacy. Data Partners within an FDN remain in control of their data, with local governance from consent for a study interest, through to the audit of its use, always respecting the local context associated with data.

## The CCV and the promise of the FDN

By design, FDNs meet the challenge of enabling access to differently located, diverse data sets for research and health system improvement. The use of the CCV model helps us to understand the sociotechnical possibilities associated with FDN in terms of their potential to enable data use without violating local norms, values, and governance arrangements, and without requiring undue work that changes the position of use within the CCV and in relation to the data subject. Unlike efforts to centralize or consolidate, an FDN maintains existing custodial and hosting relationships between data and data subjects.

As a result, FDNs are well placed to meet further challenges related to the reliability of data security and data protection in a federated system and the trustworthiness of the governance processes that constitute the system. Here, trustworthiness applies largely to the overall process where judgements about access and use are required, whereas it is reasonable to think that the security of data is a matter of reliability or assurance (Sheehan et al., 2020). In an FDN, data are held by the “controller” at the local point of origin rather than being moved, either to a different location or being shared with the researchers who are using it. The controller at the data source and their processes for making judgements thus remain the final arbiter on the use of data, so there is no change in the relationships within the system: the local data controllers have not betrayed any trust by being part of the FDN when their governance arrangements permit them to do so. Similarly, data continue to be held as securely as the local infrastructure will allow, and participation as part of the FDN does not affect this. In both cases, by preserving local relationships between the data subject and the data controller, the FDN benefits from established systems of security and trustworthiness.

## Discussion: Confronting challenges

By approaching FDNs through the lens of the CCV, it is possible to see not only how federated networks offer an opportunity for the use of data in learning health systems, but why. Specifically, we suggest, they enable data use at scale by respecting the integrity of specific socio-technical configurations of regulation, governance, and social relations (the “circles”). However, this same respect for existing arrangements presents at least two challenges for FDNs in the present and possibly in the future.

The first challenge broadly fits into the category of “return of results.” The responsibilities associated with the return of both study-relevant and incidental findings are increasingly recognized in ethical and regulatory guidance (National Academies of Sciences, Engineering, and Medicine, 2018; Thorogood et al., 2019). However, this suggests a direct relationship between researchers and patients or research participants that is a challenge for research conducted on patient data or samples ‘at a distance' from the data subject themselves and their immediate therapeutic interest. When data are aggregated, as a data lake, the responsibilities of centralized data holders related to this question might be established within the process of data consolidation, for example within the consent discussion. In the absence of such direct interactions with data subjects, FDNs need to consider how to manage these findings while protecting the integrity of each circle – including local legal and ethical frameworks for return of results - and develop carefully considered, adaptable policies that can accommodate a range of different situations and approaches.

The second challenge, one of inclusion and fairness, arises from the structure and organizational model. Some locations which have greater restrictions on data use and access, or that do not have resources to enable them to connect to the network (for example through adopting a common data model) may be excluded from the network or from specific kinds of research within the network. It is important for FDNs to be aware of those locations that are difficult to access and groups of patients who are consequently disadvantaged and, where possible, endeavor to compensate for this disadvantage. In Europe, this suggests the need to consider how FDNs are shaped by differences in data contexts associated with the divergent national appropriation of GDPR (Hansen et al., 2021). FDNs are positioned to cope with this problem by managing the existing lack of harmonization between regions. However, any forward-looking approach must be able to cope with, or even encourage, technical and social harmonization by changing, revisiting, and renewing boundaries of access and use.

Recognizing that the challenges associated with health data sharing are both social and technical, and that they relate, in large part, to the local nature of data and the form of the connection with the data subject is a beginning, but there remains hard work to be done. By involving patients, participants and the public across the network and at specific locales alongside researchers and clinicians and data controllers, divergent regions may move toward understanding the source and scale of differences and align standards and norms in ways that facilitate the movement of research through data contexts, meaning that more research can be conducted more efficiently. In this respect FDNs are well placed to instigate change and, in particular, move toward the harmonization of approaches to consent, governance and regulation while being respectful of local variation and values.

## Data availability statement

The original contributions presented in the study are included in the article/supplementary material, further inquiries can be directed to the corresponding author.

## Author contributions

RM and MS wrote the first draft of the manuscript. All authors contributed to conception and design of the perspective, contributed to manuscript revision, read, and approved the submitted version.

## Funding

This work was funded by the European Union's Horizon 2020 research and innovation programme and EFPIA, Grant number 806968. RM's contribution was also supported by Wellcome Trust Grant number 108413/A/15/D. The APC was funded by Janssen Research and Development, LLC. MS is grateful for the support of the Oxford NIHR Biomedical Research Centre.

## Conflict of interest

Author RM is an employee of Wellcome Connecting Science, part of Genome Research Limited, funded by the Wellcome Trust. Author BB is an employee of EFPIA, which is a representative organization of the pharmaceutical industry. Author AM-U is an employee of Farmaindustria, which is a representative organization of the pharmaceutical industry established in Spain. Authors GS and NH are employees of Janssen and own stock in Johnson & Johnson. Through their contribution, Janssen Research and Development, LLC was involved in the study design, collection, analysis, interpretation of data, the writing of this article or the decision to submit it for publication. The remaining authors declare that the research was conducted in the absence of any commercial or financial relationships that could be construed as a potential conflict of interest.

## Publisher's note

All claims expressed in this article are solely those of the authors and do not necessarily represent those of their affiliated organizations, or those of the publisher, the editors and the reviewers. Any product that may be evaluated in this article, or claim that may be made by its manufacturer, is not guaranteed or endorsed by the publisher.
